# Association between homocysteine levels and cognition in late-life depression

**DOI:** 10.3389/fpsyt.2025.1599716

**Published:** 2025-08-14

**Authors:** Ning Fan, Qi Zhang, Wenxuan Zhao, Yajun Yun, Meng Zhang, Yongqian Wang, Xinrui Wang, Xufang Sang, Bo Zhou, Huimei An, Fengmei Fan, Xiaole Han, Fude Yang, Luyuan Bai

**Affiliations:** ^1^ Peking University Huilonguan Clinical Medical School, Beijing Huilongguan Hospital, Beijing, China; ^2^ Wuxi Mental Health Center, Wuxi, China

**Keywords:** late-life depression, cognitive impairment, homocysteine, elderly population, depression

## Abstract

**Background:**

Cognitive impairment frequently occurs in patients with late-life depression (LLD) and could be associated with variations in homocysteine (Hcy) levels. This study aimed to evaluate the relationship between Hcy levels and cognitive function, with particular attention on how baseline cognitive status may impact this relationship.

**Methods:**

This cross-sectional study included 60 patients with LLD meeting Diagnostic and Statistical Manual of Mental Disorders, V Edition (DSM-5) diagnostic criteria and 46 age-matched healthy controls (HCs). Participants were excluded if they had severe physical illnesses. Cognitive function was assessed using the Repeatable Battery for the Assessment of Neuropsychological Status (RBANS) scale and Mini-Mental State Examination (MMSE). Hcy levels were determined.

**Results:**

Compared to HCs, LLD patients demonstrated significant impairment across all RBANS subdomains except language (*p* < 0.001), with elevated Hcy levels (*t* = 2.688, *p* = 0.008). Hcy was negatively correlated with cognition, and there was possible evidence of an interaction between Hcy and depression severity, such that this association intensified as depression severity increased (interaction *β* = 1.385, 95% confidence interval: 0.006–0.589, *p* = 0.046).Subgroup analysis showed that the negative correlation between Hcy and cognition was exclusively observed in the N-MMSE group (Normal MMSE scores, ≥26; *p* < 0.05).

**Limitations:**

The small sample size and lack of ethnic diversity may limit the generalizability of our results.

**Conclusion:**

Patients with LLD often exhibit cognitive impairment and elevated Hcy levels. Notably, the association between Hcy and cognitive function is influenced by the patients’ baseline cognitive status. This study offers novel insights into the mechanisms underlying cognitive impairment in patients with depression.

## 1 Introduction

Major depressive disorder (MDD) is common in older adults worldwide. Over 264 million people are affected by depression, which is a major public health concern (1). MDD in older people, also named late-life depression (LLD), is a chronic psychological disorder with typical clinical features (i.e., persistent depression, loss of interest in enjoyment, reduced energy, disturbed sleep, reduced concentration, and thoughts or acts of self-harming or suicide) (2).

Cognitive impairment is a common and persistent symptom in patients with LLD (3). Patients with depression often exhibit reduced efficiency in attention and executive functions compared to healthy participants (4). The acute phase of LLD in patients with depressive disorders frequently involves cognitive dysfunction, including a decline in executive functioning, verbal fluency, working memory, and attentional control (3, 5). Approximately 30–50% of patients with LLD can improve their mood with antidepressants; however, some of them have cognitive deficits (6). Cognitive impairment in patients with LLD can also lead to unfavorable outcomes with antidepressants (7), such as poor and slow response to pharmacotherapy (7) and increased risk of suicide and functional disability (2). Certain cognitive impairments, especially executive dysfunction, have a substantial clinical impact, frequently contributing to poor therapeutic outcomes (8). A previous study reported that participants who performed markedly poorer in executive function tests responded to anti-depressive therapy in a more satisfactory manner than those who obtained better scores (9).

Impaired cognitive function in patients with LLD and its relationship with dementia is an ongoing debate regarding the extent to which these impairments are due to emotional symptoms, causing worse cognitive performance (10). Mild cognitive impairment during episodes of major LLD does not progress to dementia. However, older patients with major depression and serious cognitive dysfunction are at a high risk of developing Alzheimer’s disease (11). Patients with both cognitive dysfunction and late-onset depression may already have early-stage dementia (6). Therefore, cognitive dysfunction in patients with depression has heterogeneous etiologies and outcomes (7), and elucidating the pathophysiological mechanisms of the cognitive impairment associated with LLD is essential.

Homocysteine (Hcy), an intermediate in the methionine cycle, plays a crucial role in various biological processes and has been linked to the development of depression (12), vascular disease, and cognitive compromise (13). The metabolic process of Hcy depends on its enzymatic action and synergistic involvement of essential cofactors such as folate and B vitamins, including B6 and B12 (13). Hcy homeostasis can also be influenced by genetic factors, such as polymorphisms in the *MTHFR* gene, which encodes an enzyme involved in Hcy metabolism. Patients with elevated Hcy levels have reduced blood folate, vitamin B12, and vitamin B6 concentrations, as well as alterations in *MTHFR* polymorphisms (14). Several pathogenic mechanisms have been proposed to explain the relationship between Hcy levels and depression or dementing disorders (15) and elevated Hcy levels and low baseline folate, vitamin B12, and vitamin B6 concentrations could be used to predict the development of depression (15). Some studies have found that depressed patients with elevated Hcy exhibit a reduction in Hcy levels following supplementation with folate, vitamin B6, and vitamin B12 supplementation, without a corresponding improvement in depressive symptoms (16). In contrast, other studies suggest that such supplementation does alleviate depressive symptoms (17, 18), resulting in inconsistent findings across the literature. Increased Hcy levels have also been associated with cognitive impairment (19). Higher Hcy levels are associated with poor functioning across several cognitive domains (17). The observation that treatment of patients with cognitive dysfunction with folate and vitamin B12 decreases Hcy levels and improves cognitive function (20) suggests that Hcy is a crucial modifiable cause of cognitive impairment in patients with LLD.

A previous study (21) investigated possible associations between Hcy levels and cognitive screening test scores in patients with geriatric depression and observed these relationships in older patients with depression and without concomitant vascular diseases. Higher plasma Hcy levels were correlated with poorer cognitive performance, but the neuropsychological assessment was limited to the Mini-Mental State Examination (MMSE) and the sample size was small (22). Significant associations between Hcy levels and severe cognitive impairment in patients with geriatric depression were recently reported, providing evidence for the association of this index with cognitive dysfunction, often accompanied by LLD. Interestingly, older adults with high Hcy concentrations and LLD had more severe cognitive impairment than those with LLD or high Hcy concentrations alone. However, no subcategories of LLD based on specific characteristics exist (21).

Few research groups have documented Hcy abnormalities in LLD, and their relationship with cognitive impairment remains unknown. Furthermore, previous studies evaluated patients as a single cohort without considering individual cognitive baselines and often used rudimentary tools for cognitive assessment. Therefore, this study aims to fully consider different baseline cognitive levels and thoroughly explore the relationship between Hcy, late-life depression, and cognitive impairment, with the goal of filling the knowledge gap regarding potential differences in homocysteine levels among elderly patients with late-life depression.

## 2 Methods

### 2.1 Patients

Patients with LLD were recruited from the Department of Beijing Huilongguan Clinical Medical School, Peking University, PR, China. All patients met the following inclusion criteria: (1) 60–85 years old; (2) newly admitted, met the criteria of the Diagnostic and Statistical Manual of Mental Disorders, V Edition (DSM-5) for unipolar MDD, had a score >17 in the 17-item Hamilton Depression Rating Scale (HAMD-17), and Clinical Dementia Rating (CDR) <0.5; (3) received no antidepressant therapy for at least 2 weeks; (4) had at least 5 years of formal education; and (5) Han ethnicity and right-handedness. The exclusion criteria were as follows: (1) history of other psychiatric disorders before depression onset; (2) history of severe medical illness, severe brain injury, epilepsy, cerebrovascular disease, or long-term neuropsychiatric complications such as tumors; (3) history of head trauma, Parkinson’s disease, or multiple sclerosis; and (4) alcohol or drug abuse. Healthy volunteers were recruited from local communities through advertisements. The inclusion criteria for all healthy controls (HCs) were as follows: (1) No serious mental illness according to the DSM-5 criteria; (2) no severe medical illness; (3) no history of neurological disorders or MRI evidence of structural brain abnormalities; and (4) no family history of psychiatric disorders.

The Ethics Committee of Beijing Hui Long Guan Hospital approved this study. Informed consent was obtained from all patients.

### 2.2 Clinical symptoms and cognitive function assessments

Depressive symptoms were measured using the HAMD-17 score, and the Hamilton Anxiety Scale (HAMA) was used to assess anxiety and depression symptoms.

The MMSE, a widely used screening instrument administered by trained researchers, provides a rapid initial assessment of global cognitive function. It evaluates cognitive domains through 19 items, with a total score of 30 points. A score of ≥26 indicates normal cognition, while that of <26 suggests cognitive impairment. The Repeatable Battery for the Assessment of Neuropsychological Status (RBANS), a standardized measurement tool that includes five subscales (immediate memory, delayed memory, attention, language, and visuospatial function), has good internal consistency and acceptable reliability in Chinese patients with MDD (23). Therefore, the RBANS was used to identify and characterize cognitive decline in patients with depressive disorders.

### 2.3 Blood sample collection and plasma Hcy level measurement

Venous blood (4 mL) was collected from each patient between 06:00 AM and 07:00 AM after 12 h of fasting. Hcy concentrations were quantified enzymatically on a Beckman Coulter AU5800 analyzer. Reagent blank absorbance exceeded 0.80; intra-assay CV (10 replicates) was ≤5%; analytical sensitivity (absolute ΔA at 10 μmol/L) was >0.2.

### 2.4 Statistical analyses

SPSS 22.0 software (SPSS Inc., Chicago, IL, USA) was used to analyze demographic variables, including age, body mass index, and sex. Qualitative variables are presented as frequencies (counts) and percentages, while quantitative variables are reported as means ± standard deviations. Chi-squared tests compared dichotomous variables between groups, and independent sample t-tests or one-way ANCOVAs analyzed continuous variables. Bonferroni correction adjusted for multiple comparisons across RBANS subscales.

Pearson’s correlation analysis examined the relationship between Hcy levels and clinical parameters. A multiple linear regression model with interaction terms assessed whether HAMD modified the effect of Hcy on cognitive function (total RBANS score). The model included main effects of Hcy, HAMD, and their interaction (Hcy × HAMD), with age, education, and gender adjusted as covariates. Collinearity was evaluated via variance inflation factors, and interaction term significance was tested using t-statistics. Mediation analysis via Bootstrap resampling (5,000 iterations) explored the mechanism between Hcy and MMSE using “Immediate Memory” and “Delayed Memory” score grouping as the mediator. Statistical significance was defined as p < 0.05.

## 3 Results

### 3.1 Demographic information and clinical data of patients and HCs

Sixty patients with LLD and 46 HCs were eligible for inclusion. The demographic and clinical characteristics of the participants are presented in **Table 1**. No significant differences were observed between the LLD and HC groups regarding age, sex and other demographic characteristics (all *p* > 0.05) (**Table 1**).

**Table 1:** Demographic information of patients with late-life depression and healthy controls.

Clinical information	N-MMSE N=24	L-MMSE N=36	LLD N=60	HC N=46	LLD vs. HC *t/χ^2^*	*p*	N/L-MMSE vs. HC *F/t/χ^2^*	*p*
Age (years)	69.38 ± 16.36	69.52 ± 6.96	69.94 ± 11.33	67.70 ± 4.36	1.274	0.205	0.482	0.619
Sex (male/female)	7/17	12/24	29/41	16/30	0.152	0.426	0.227	0.893
Education	11.67 ± 4.10	9.89 ± 3.62	11.08 ± 3.63	11.72 ± 3.51	-0.929	0.329	2.511	0.086
Height (cm)	161.74 ± 8.40	162.72 ± 8.43	162.39 ± 8.33	163.83 ± 6.87	-0.869	0.387	0.502	0.607
Weight (kg)	62.54 ± 12.22	61.18 ± 10.77	61.54 ± 11.14	66.83 ± 8.20	-2.455	0.016	2.842	0.064
Smoking (Yes/No)	21/3	34/2	55/5	42/4	5.135	0.077	1.620	0.445
Drinking (Yes/No)	22/2	33/3	55/5	41/5	2.141	0.343	0.703	0.704
Hcy levels	12.46 ± 3.64	16.77 ± 6.05	15.51 ± 5.98	12.64 ± 3.70	2.688	0.008	8.560	<0.001
Age of onset (years)	65.74 ± 14.50	57.19 ± 14.07	61.06 ± 14.48	—	—	—	2.248	0.028
Family history	4	12	16	—	—	—	3.314	0.191
HAMD	24.54 ± 8.83	25.83 ± 7.42	25.53 ± 8.12	—	—	—	0.612	0.543
HAMA	22.50 ± 11.24	21.42 ± 8.72	21.98 ± 10.03	—	—	—	0.420	0.676

Hcy, homocysteine; MMSE, Mini-Mental State Examination; L-MMSE, patients with a low MMSE score (<26). N-MMSE: patients with a normal MMSE score (≥26); LLD, late-life depression. HC, healthy controls; HAMA, Hamilton Anxiety Scale; 17-HAMD, 17-item Hamilton Depression Rating Scale.

### 3.2 Cognitive characteristics of patients and controls

The mean MMSE total score in patients with depression was lower than that in the HC group (*p* < 0.001). Overall, 60% of the patients with depression had cognitive impairment, defined as an MMSE score <26. Compared with the HCs, all patients showed some cognitive deficits in the overall RBANS subdomains, except for the language score (all *p* < 0.05) (**Table 2**).

**Table 2:** Cognitive characteristics of patients and healthy controls.

Clinical information	N-MMSE	L-MMSE	LLD	LLD vs. HC *t*	*p*	HC	N/L-MMSE *F*	*p*
MMSE score	26.67 ± 3.41	20.39 ± 5.64	22.87 ± 5.70	-5.941	<0.001	27.96 ± 1.23	20.626	<0.001
Immediate Memory	24.67 ± 8.73	18.53 ± 8.79	20.11 ± 9.71	-8.723	<0.001	35.74 ± 8.61	9.311	0.003
Visuospatial	28.17 ± 7.79	22.92 ± 11.57	24.25 ± 11.21	-4.153	<0.001	31.46 ± 4.19	4.647	0.035
Language	23.42 ± 6.61	19.33 ± 6.51	20.25 ± 7.54	-0.654	0.514	21.02 ± 3.12	5.519	0.022
Attention	26.04 ± 13.37	21.83 ± 13.23	22.86 ± 13.73	-9.533	<0.001	47.24 ± 12.51	12.429	<0.001
Delayed Memory	28.67 ± 10.51	22.72 ± 9.05	23.95 ± 10.82	-10.46	<0.001	43.39 ± 7.61	4.265	0.043
Total RBANS score	130.96 ± 32.31	105.33 ± 42.48	111.42 ± 44.44	-9.024	<0.001	178.85 ± 28.65	6.334	0.015

MMSE, Mini-Mental State Examination; RBANS, Repeatable Battery for the Assessment of Neuropsychological Status; LLD, patients with late-life depression; HC, healthy controls; L-MMSE, patients with a low MMSE score (<26). N-MMSE, patients with a normal MMSE score (≥26).

The patients with LLD were further divided into two groups according to their MMSE scores. The first group included patients with a low MMSE score (<26; L-MMSE) and second group included those with a normal MMSE score (≥26; N-MMSE). No significant differences in age, sex, height, weight, or HAMD-17 or HAMA scores were observed between the two groups (*p* > 0.05) (**Table 1**).

Compared to the HCs, significantly worse performance was observed in terms of immediate memory, attention, delayed memory, and the total scale score in RBANS in the N-MMSE group (*p* < 0.05). In addition, significantly greater cognitive impairment was observed in the L-MMSE group compared to both HC and N-MMSE groups across all domains (all *p* < 0.05; subscale-specific differences detailed in **Table 2**).

### 3.3 Differences in Hcy levels between patients with LLD and HCs

**Figure 1:**
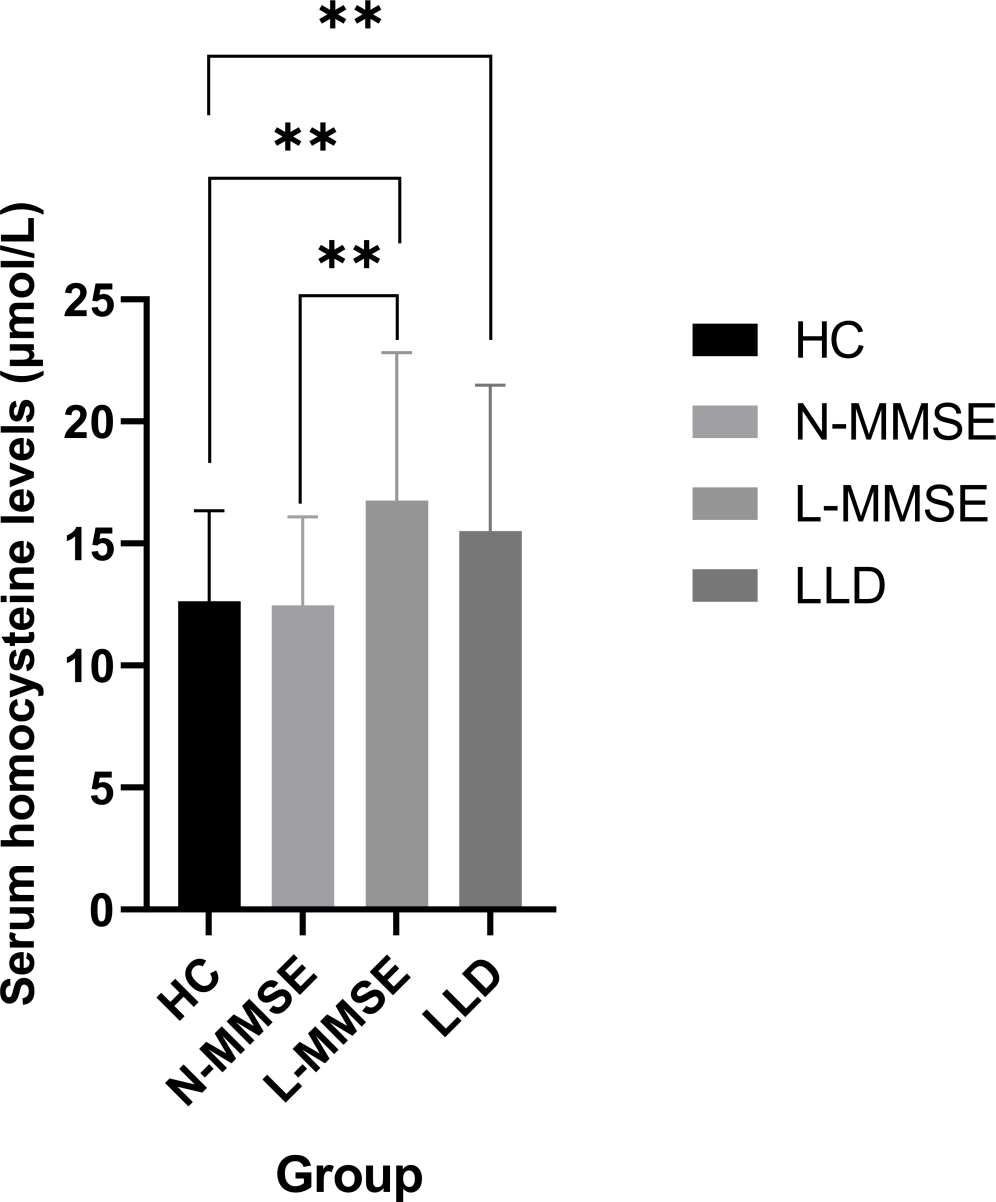
Compared to the HC group, LLD group and L-MMSE group has elevated Hcy levels (all p < 0.01**). Compared to the N-MMSE group, L-MMSE group has elevated Hcy levels (p < 0.01**). HC: healthy controls; LLD: late-life depression; L-MMSE: patients with a low MMSE score (<26); N-MMSE: patients with a normal MMSE score (≥26). MMSE: Mini-Mental State Examination.

Elevated Hcy levels were observed in patients with LLD (F = 8.560, *p* < 0.001) (**Table 1**), which were dependent on group allocation per MMSE score. In particular, Hcy levels in patients in the L-MMSE group were higher than those in N-MMSE group and HCs (*p* < 0.05). No significant differences in Hcy levels were observed between patients in the N-MMSE group and HCs (*p* > 0.05) (**Table 1**; **Figure 1**).

### 3.4 Association between Hcy levels and cognitive function

**Figure 2:**
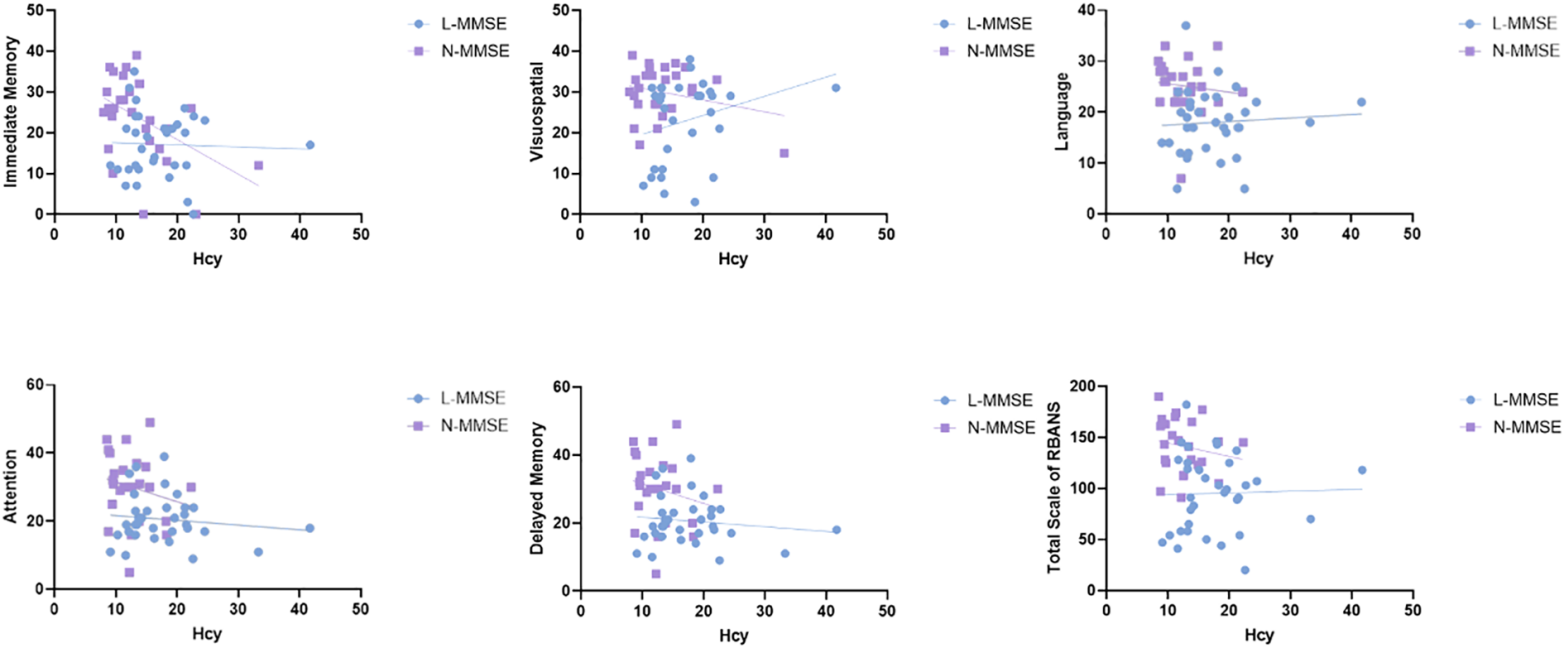
Relationship between Hcy levels and cognitive impairment in patients with LLD and HCs. Hcy level is significantly negatively associated with MMSE scores (*r* = -0.286, *p* = 0.029), immediate memory(*r* = -0.323, *p* = 0.01), delayed memory (*r* = -0.336, *p* = 0.008), and the total RBANS scale score (*r* = -0.270, *p* = 0.034) in patients with LLD. However, no significant correlation is observed between Hcy levels and cognitive functions in the HC group (all *p* > 0.05). HC, healthy controls; Hcy, homocysteine; LLD, patients with late-life depression; MMSE, Mini-Mental State Examination; RBANS, Repeatable Battery for the Assessment of Neuropsychological Status.

In the patients with LLD, Hcy levels were significantly negatively associated with MMSE scores(*r* = -0.286, confidence interval [CI]: -0.496–0.073, *p* = 0.029), immediate memory (*r* = –0.283, 95%CI: -0.497–0.106, *p* = 0.031), delayed memory (*r* = -0.279, 95%CI: -0.480–0.059, *p* = 0.003), and the total RBANS scale score (*r* = −0.270, 95%CI: -0.470–0.022, *p* = 0.034). No significant correlation was observed between Hcy levels and cognitive function in the HC group (all *p* > 0.05) (**Figure 2**). We further performed multiple linear regression analysis to adjust for confounding variables such as age, sex, and education. The results showed that after adjusting for confounding factors, serum Hcy levels were independently and negatively correlated with RBANS scores (*β* = -0.311, 95%CI: -4.310 to -0.310, *p* = 0.024). Further inclusion of an interaction term between Hcy and HAMD significantly improved the model’s goodness of fit (*ΔR²* = 0.058, *p* = 0.019). The interaction effect test revealed that the impact of Hcy on cognitive function was increased with the severity of depression (*β* = 1.385, 95%CI: 0.006–0.589, *p* = 0.046) (**Table 3**).

**Table 3:** Multivariate linear regression model for homocysteine and cognitive in patients with later-life depression.

Independent variable	Without interaction terms				Including interaction terms			
	B	β	95%CI	*P*	B	β	95%CI	*P*
Age	-0.869	-0.225	-1.898–0.161	0.096	-1.344	-0.347	-2.448–0.239	0.018
Education	-2.719	-0.237	-5.635–0.198	0.067	-2.964	-0.259	-5.813–0.116	0.042
Gender	12.449	0.132	-12.453–37.352	0.321	14.169	0.150	-10.124–38.462	0.247
Hcy	-2.310	-0.311	-4.310–0.310	0.024	-4.447	-0.824	-8.806–0.088	0.046
HAMD	0.236	0.044	-1.199–1.671	0.743	5.709	0.768	-2.391–13.809	0.163
Hcy×HAMD	–	–	–	–	0.298	1.385	0.006–0.589	0.046

CI, confidence interval; Hcy, homocysteine; HAMD, Hamilton Depression Rating Scale.

**Figure 3:**
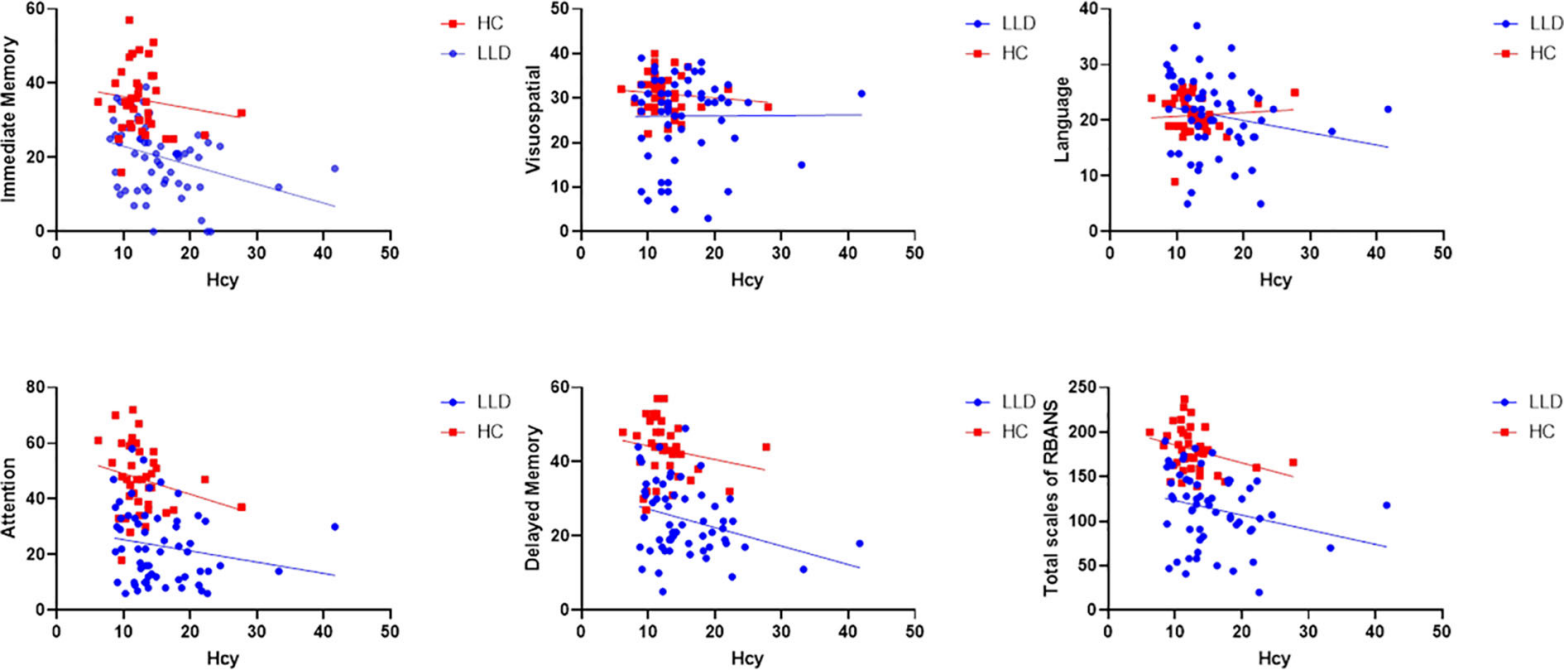
Relationship between Hcy levels and cognitive impairment in the N-MMSE and L-MMSE groups. In the N-MMSE group, Hcy levels are negatively associated with MMSE scores (*r* = -0.587, *p* = 0.003), attention (*r* = -0.415, *p* = 0.049), delayed memory (*r* = -0.419, *p* = 0.046), and the total RBANS scale score (*r* = -0.499, *p* = 0.015). However, Hcy levels are not associated with RBANS scores for immediate memory (*r* = -0.409, *p* = 0.052), language (*r* = -0.282, *p* = 0.193), or visuospatial function (*r* = -0.321, *p* = 0.126). Hcy levels are also not correlated with any index of cognitive function, including MMSE score, immediate memory, delayed memory, attention, language, visuospatial function, and the total RBANS scale score in the L-MMSE group (all *p* > 0.05). Hcy, homocysteine; L-MMSE, patients with a low MMSE score (<26); MMSE, Mini-Mental State Examination; N-MMSE, patients with a normal MMSE score (≥26); RBANS, Repeatable Battery for the Assessment of Neuropsychological Status.

**Figure 4:**
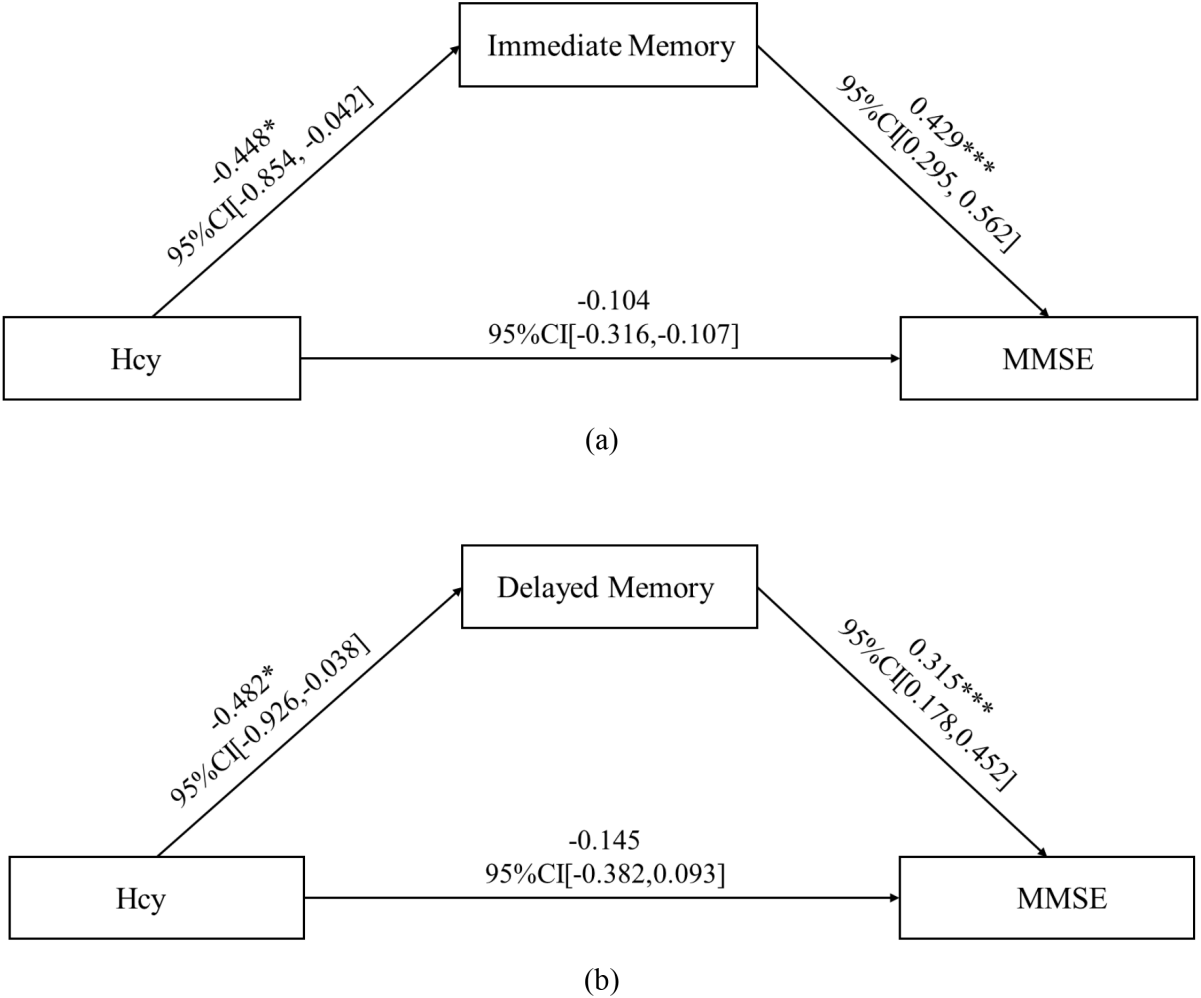
Mediation path coefficients for Hcy, immediate memory/delayed memory, and MMSE. Direct effect of Hcy on MMSE is nonsignificant (*t* = -1.221, *p* = 0.227), with Hcy significantly predicting immediate memory (*t* = -2.174, *p* = 0.038), and delayed memory (t = 4.593, *p* < 0.001) significantly predicting MMSE, and the Bootstrap-derived indirect effect of -0.192 (95%CI: -0.479, -0.052) and -0.152 (95%CI: -0.362, -0.035) excluding zero, indicating full mediation by immediate memory and delayed memory. CI, confidence interval; Hcy, homocysteine; MMSE, Mini-Mental State Examination.* *p* < 0.05; ***p < 0.001.

In the subgroup analysis, Hcy levels in the N-MMSE group were negatively associated with MMSE scores (*r* = -0.587, *p* = 0.003), attention (*r* = -0.415, *p* = 0.049), delayed memory (*r* = -0.419, *p* = 0.046), and the total RBANS scale score (*r* = -0.499, *p* = 0.015). No significant associations were observed in the L-MMSE group (all *p* > 0.05) (**Figure 3**). Further, we conducted mediation analysis using immediate memory and delayed memory as mediating variables, and found that the direct effect of Hcy on MMSE was nonsignificant (*t* = -1.221, *p* = 0.227), with Hcy significantly predicting immediate memory (*t* = -2.174, *p* = 0.038), and delayed memory (*t* = 4.593, *p* < 0.001) significantly predicting MMSE, and the Bootstrap-derived indirect effect of -0.192 (95%CI: -0.479, -0.052) and -0.152 (95%CI: -0.362, -0.035) excluding zero, indicating full mediation by immediate memory and delayed memory (**Figure 4**).

## 4 Discussion

This study investigated the relationship between Hcy levels and cognitive function in patients with LLD according to their MMSE scores. Cognitive function, as measured by RBANS scores, differed across groups. The L-MMSE group exhibited the poorest cognitive performance, followed by the N-MMSE group; the HC group showed the best cognitive function. Hcy levels were higher in patients with LLD than in HCs, whereas they were significantly increased only in the L-MMSE group when the subgroups were assessed. Interestingly, Hcy levels were correlated with cognitive impairment in the N-MMSE group but not in L-MMSE group.

### 4.1 Depression and cognitive impairment

In this study, the cognitive performance—including immediate memory, visuospatial function, attention, and delayed memory—in patients with LLD was lower than that in healthy older individuals. This finding confirms previous reports on the presence of mild-to-moderate cognitive dysfunction in patients with LLD (6, 8, 9, 24). For example, a previous study reported that individuals with depression exhibited significant attention impairments, with reduced ability to sustain focus and filter out distractions (4). Memory deficits, particularly regarding episodic and working memory, have also been frequently reported (24). Older adults with depression performed poorly in recalling recent events and manipulating information in short-term memory (8). Executive function, which encompasses skills such as planning, problem-solving, and cognitive flexibility, is also compromised in patients with LLD. Furthermore, older adults with depression struggle with tasks that require strategic decision-making and organization of complex information (6). Paelecke-Habermann (25) summarized attention, executive function, and memory deficits after remission, and found that reduced depressive symptoms did not necessarily lead to cognitive improvement, possibly because cognitive impairment is a trait marker rather than a state marker of depression. These impairments may be attributed to several factors. One possible mechanism underlying cognitive impairment in LLD is the dysregulation of the system of Hcy metabolism (21). Changes in Hcy levels have been implicated in depressive symptoms and cognitive dysfunction (20, 21).

### 4.2 Depression and Hcy levels

In this study, Hcy levels were elevated in older patients with depression, consistent with the results of previous research. Older adults with depression have higher Hcy levels than their healthy counterparts (13). Large meta-analyses have shown increased serum and plasma Hcy levels in patients with MDD compared to healthy individuals (13). Compared with MDD, LLD is characterized by less prominent affective symptoms, obvious somatic symptoms, and more severe cognitive impairment (2, 8, 9). Whether regarded as a special subtype of MDD or an independent diagnostic entity, LLD has been considered in previous studies to have specificity in its pathogenesis and related biological manifestations (6). For example, some studies suggest that the role of Hcy in the pathogenesis of MDD may be related to metabolites of monoamine neurotransmitters, including serotonin, dopamine, and norepinephrine (26), while the association between Hcy and the pathogenesis of LLD is more influenced by organic factors such as cerebrovascular conditions. As shown in some studies, elevated Hcy levels have been associated with vascular endothelial dysfunction and increased oxidative stress, both of which contribute to neuroinflammation and neuronal damage, thereby influencing mood regulation (27, 28).

### 4.3 Relationship between Hcy levels and cognitive impairment in patients with LLD

The current study specifically highlights the connection between elevated Hcy levels and compromised cognitive function in patients with LLD. Cognitive performance and Hcy levels differed in the L-MMSE and N-MMSE groups. Notably, Hcy levels were significantly increased in the L-MMSE group but correlated with cognitive impairment in the N-MMSE group.

This seemingly paradoxical phenomenon may be related to a “threshold-dependent neurotoxicity mechanism” of Hcy. The role of NMDA receptors in cognitive impairment is well established[]. Research indicates that Hcy has different effects on NMDA receptors depending on its concentration (29). Studies by Bolton et al. showed that Hcy within the physiological concentration range increases the peak current of NMDA receptors, reduces receptor desensitization, and enhances glutamatergic transmission (30). Furthermore, Hcy can act as an agonist or co-agonist at NMDA receptors, increasing the excitability of glutamatergic neurons (30). In contrast, at higher concentrations (>15 μmol/L), Hcy can rapidly amplify its own concentration through an “auto-sensitization response.” High concentrations of Hcy exert neurotoxic effects. It interacts with the glutamate/cystine transporter (also known as the Xc- transport system), leading to activation of adjacent ionotropic glutamate receptors. This causes excessive NMDA receptor activation, resulting in excitotoxicity, disruption of synaptic formation, loss of prepulse inhibition, and ultimately, neuronal death (31). At this stage, neuronal compensatory mechanisms fail. This may explain why the linear association disappears in patients with severe cognitive impairment (L-MMSE group). It also indicates that the same metabolic indicators may exert distinct effects on diseases, depending on the underlying disease state. As shown in a large-sample study: analysis of NHANES data revealed a positive correlation between HDL levels and cognitive function, yet this protective effect of HDL on cognition was absent in individuals with depressive symptoms (32). This phenomenon further underscores the complex interplay between physiological indicators and disease processes.

## 5 Limitations

First, because this is a cross-sectional study, it cannot fully rule out the possibility that some participants may represent prodromal stages of dementia. These individuals might ultimately progress to dementia, which could potentially influence the study outcomes. Second, this study has a relatively small sample size, which may have compromised the statistical power to detect subtle associations and the stability of regression estimates. This could potentially affect the generalizability of our findings, particularly regarding the precision of effect size estimates for the association between Hcy levels and cognitive function. Future research with a larger, more diverse sample is warranted to validate our results and enhance the robustness of the conclusions, especially in systematically exploring the impact of confounding factors and long-term outcomes. Third, our recruitment of “newly admitted” participants specifically targeted inpatients receiving standardized care protocols at tertiary psychiatric hospitals. While this enhanced internal consistency in treatment exposure, it may limit generalizability to outpatient or community-dwelling populations with LLD. Fourth, the restriction to right-handed Han Chinese participants was implemented to control for neuroanatomical lateralization confounds and genetic heterogeneity. However, this precludes extrapolation of findings to left-handed individuals or diverse ethnic groups, warranting validation in multiethnic cohorts.

## 6 Conclusion

Our study found that patients with LLD experienced cognitive deficits alongside elevated Hcy levels. Specifically, in these patients, Hcy was negatively correlated with cognitive function; the strength of this association was related to patients’ baseline cognitive status, with depression severity possibly exerting an interactive effect. This study contributes to the growing body of evidence suggesting a significant correlation between elevated Hcy levels and cognitive functional impairment in LLD based on MMSE scores. Based on these findings, future studies should use a longitudinal design to monitor the temporal sequence of changes in Hcy levels, cognitive function, and depressive symptoms; this approach could help clarify whether treatment resolves symptoms and whether cognitive function can be restored in both groups, irrespective of MMSE scores, thus enhancing the robustness of our results. If future studies confirm the association between Hcy levels and potential to reverse cognitive dysfunction, then lowering Hcy levels through vitamin and folic acid supplementation would likely represent a modifiable risk factor for cognitive impairment in older adults with depression.

## Data Availability

The raw data supporting the conclusions of this article will be made available by the authors, without undue reservation.
